# Exploring the Impact of Tensions in Stakeholder Norms on Designing for Value Change: The Case of Biosafety in Industrial Biotechnology

**DOI:** 10.1007/s11948-023-00432-6

**Published:** 2023-03-07

**Authors:** Enrique Asin-Garcia, Zoë Robaey, Linde F. C. Kampers, Vitor A. P. Martins dos Santos

**Affiliations:** 1 Laboratory of Systems and Synthetic Biology, Wageningen University & Research, 6708 WE Wageningen, The Netherlands; 2 Bioprocess Engineering Group, Wageningen University & Research, 6700 AA Wageningen, The Netherlands; 3 Department of Social Sciences, Wageningen University & Research, 6708 WE Wageningen, The Netherlands; 4 LifeGlimmer GmbH, Berlin, Germany

**Keywords:** Biosafety, Value sensitive design, Genetic safeguards, Multistakeholder approach, Industrial biotechnology, Safe-by-design framework

## Abstract

Synthetic biologists design and engineer organisms for a better and more sustainable future. While the manifold prospects are encouraging, concerns about the uncertain risks of genome editing affect public opinion as well as local regulations. As a consequence, biosafety and associated concepts, such as the Safe-by-design framework and genetic safeguard technologies, have gained notoriety and occupy a central position in the conversation about genetically modified organisms. Yet, as regulatory interest and academic research in genetic safeguard technologies advance, the implementation in industrial biotechnology, a sector that is already employing engineered microorganisms, lags behind. The main goal of this work is to explore the utilization of genetic safeguard technologies for designing biosafety in industrial biotechnology. Based on our results, we posit that biosafety is a case of a changing value, by means of further specification of how to realize biosafety. Our investigation is inspired by the Value Sensitive Design framework, to investigate scientific and technological choices in their appropriate social context. Our findings discuss stakeholder norms for biosafety, reasonings about genetic safeguards, and how these impact the practice of designing for biosafety. We show that tensions between stakeholders occur at the level of norms, and that prior stakeholder alignment is crucial for value specification to happen in practice. Finally, we elaborate in different reasonings about genetic safeguards for biosafety and conclude that, in absence of a common multi-stakeholder effort, the differences in informal biosafety norms and the disparity in biosafety thinking could end up leading to design requirements for compliance instead of for safety.

## Introduction

### Biotechnology, Synthetic Biology, and Biosafety

Biotechnology, and the field of synthetic biology (SynBio) in particular, have been described as promising technologies that at the same time raise concerns of uncertainty and risks to health and the environment (Andrianantoandro et al., 2006; Scientific Committee, 2020; Fröhling & Hiete, 2020; Linkov et al., 2018; Remer et al., 2001; Trump et al., 2020). In this paper, we turn our attention to engineered microorganisms in industrial biotechnology (IB), a mature sector with promises of decreasing environmental impacts (Rischer et al., 2020), positive socioeconomic effects (Lokko et al., 2018), competitive processes and products (Kiefer et al., 2021), and development in rural areas (Fröhling & Hiete, 2020; Tylecote, 2019). Developments in the last decade have superseded the initial hype brought by the onset of SynBio with impressive technological achievements (Meng & Ellis, 2020) like the synthesis of working bacterial genomes (Gibson et al., 2010), the development of computer-aided systems for logic circuit construction in bacteria (Nielsen et al., 2016), the CRISPR (acronym for clustered regularly interspaced short palindromic repeats)—Cas genome editing revolution (Jinek et al., 2012) and the use of non-natural building blocks and non-canonical chemistry that gave rise to the subfield of xenobiology (Schmidt et al., 2017). The development of these technologies has brought forth the concepts of trophic and semantic containment of engineered microorganisms; in other words, possibilities to build safety as an inherent feature of a microorganism. The idea of genetic safeguard technologies for biosafety and related possible implementations therefore emerged once again, as it had previously done in the eighties linked to the idea of uncontained genetically engineered organisms.

### Biosafety Then

Biosafety is a complex concept traditionally defined as the set of containment principles, facility design, practices and procedures to prevent occupational problems or the release of engineered organisms to a non-permissive environment (Nordmann, 2010). Other definitions of biosafety focus directly on the risks and the capability of the engineered biological agents to cause disease, of greater or lesser severity, in humans, animals and plants (Beeckman & Rüdelsheim, 2020; Kelle, 2009).

Almost 50 years ago, the Asilomar Conference set the foundations for the way recombinant DNA (acronym of deoxyribonucleic acid) is dealt with by delivering a series of principles to handle potential biohazards. These guidelines mainly focused on making containment an essential consideration in the experimental design and matching the effectiveness of such containment to the estimated risk. Moreover, it was indicated that given the difficulty of risk estimation, this would be intuitive at first and improved later as additional knowledge and technologies came into the picture. Thus, technology progress and new research developments would bring along the means to assess and balance risks with reexamined appropriate levels of containment (Berg et al., 1975).

The proposals of Asilomar were meant as a guide that included recommendations regarding, amongst others, the development of safer vectors and hosts, laboratory procedures and education and reassessment, thereby providing the means for a structured and standard approach. As a consequence, the value of biosafety today builds largely on foundations and discussions that took place in the seventies, and slowly crystallized into policies in the nineties and early two-thousands. We observe however that the principles, designs, and practices are changing, and therefore that biosafety can present a case of value change, where scientific knowledge about new ways to realize biosafety could afford new uses of engineered microorganisms and therefore the fulfillment of the benefits of new biotechnologies.

### Biosafety Now

In this section, we present new technological developments that challenge the traditional understanding of the value of biosafety, by for instance offering new possibilities in terms of designs. In recent times biosafety is attracting more attention because it may be a limiting factor in the development of advanced technologies. Taking into account the pace and progress of biotechnology and synthetic biology, a lack of international norms is apparent, as well as a dearth of guidance at some of the value levels, mostly related to the risks that have emerged as a consequence of the progress of technology (Kwik Gronvall, 2017; Kwik Gronvall & Rozo, 2015). As indicated, Asilomar principles resulted in norms that were established decades ago, but now more information related to biosafety seems to be necessary to set policy for the recent developments.

In a recent comprehensive review, Hewett et al. identified 44 discrete risks in synthetic biology: 18 of those related to human health, which were subsequently clustered into 4 main categories: allergies, antibiotic resistance, carcinogens, and pathogenicity or toxicity; and 26 related to the environment, later categorized into: change or depletion of the environment, competition with native species, horizontal gene transfer, and pathogenicity or toxicity (Hewett et al., 2016; Wang & Zhang, 2019). This points to the need for further specification of issues when speaking of the value of biosafety.

Despite international agreements such as the Cartagena Protocol on Biosafety, legal regulation differs from country to country, the EU being known for its more stringent regulation. This has led to recurrent calls to reconsider how to do biosafety (Trevan, 2015; and in the related field of biosecurity Evans et al., 2020), suggesting that the specification of biosafety as a value is not only limited to understanding risks, but also extends to how biosafety is understood. One of the recent promising trends to deal with biosafety is centered around the concept of Safe-by-design, deriving from other technological fields (Bouchaut & Asveld, 2020). Applying Safe-by-design (SbD) principles to biotechnology and SynBio offers a pre-emptive approach to risk management and aspires to minimize the risks of these technologies by making safer design choices during the early stages of the innovation trajectory, preferably at the research and development (R&D) and design phases (van de Poel & Robaey, 2017).

Genetic safeguard strategies, previously popular in the eighties and nineties, have experienced a new golden age this past decade resulting in a prolific repertoire of diverse approaches (Asin-Garcia et al., 2020; Schmidt & de Lorenzo, 2016; Whitford et al., 2018). These technological investigations have been justified as inherent safety mechanisms to control the aforementioned risks of engineered microorganisms (Schmidt, 2010), a claim that has been underlined by policy in numerous occasions (Gutmann et al., 2011; Ministerie van Volksgezondheid 2016; Scientific Committee on Health and Environmental Risks (SCHER) et al. 2015). Biosafety is at the epicenter of discussions about governance and ethics of technology (Robaey, 2018; Stemerding et al., 2019; Trump et al., 2020) and remains an essential component of the SynBio paradigm, as reflected by its importance in the International Competition on the Genetically Engineered Machine (the iGEM competition), the cradle of the synthetic biologists of the future (Guan et al., 2013; Millett et al., 2019).

Nowadays, one could argue that approaches to safety and responsibility keep partly shaping the research agenda of the biotechnology and SynBio laboratories (Aparicio, 2021). While this might be true for certain academic research projects, the situation is probably different for an already established sector like IB (Asveld et al., 2020; Fröhling & Hiete, 2020; Nuzzo et al., 2020; Straathof et al., 2019), where biosafety might be simply understood as technical compliance to the current regulations about management of genetically modified organisms (GMO). References to strategies for increasing the safety of engineered or synthetic organisms are widespread, but discussions about their use and applicability hardly ever get more specific than that. Consequently, while the appreciation of genetic safeguard technologies by policy and regulatory bodies continues developing, the formulation of such tools for real-life scenarios beyond academic research remains an unfulfilled ideal (Kallergi et al., 2021; Bouchaut & Asveld, 2021).

### Investigating a Changing Value

In this research, we investigate the tension between the potential to do biosafety differently thanks to genetic safeguard technologies described in the previous section, and the lack of change of practices for biosafety in real world IB applications. We believe that this tension is an indicative case of changing value, and more specifically value specification according to van de Poel’s taxonomy (2018). In this research, we show that value specification does not simply happen. In order to do so, we use the concept of value hierarchy, where design requirements are formulated for the sake of norms, which are in turn formulated for the sake of certain values (van de Poel, 2013). This allows us to add nuance to the phenomenon of value specification, and to explain how such value change could be supported. In some cases, like for the value of biosafety, that could be beneficial to society at large by moving away from a mentality of compliance, and increasing biosafety barriers in IB.

In addition, because the first author of this paper is a bioengineer working on the design of said genetic safeguards in IB, we organize our investigation following Value Sensitive Design (VSD), a framework that lends itself to understanding scientific and technological choices in their social context. VSD invites considering the role of the designer when formulating design requirements that can answer to given norms to fulfill stakeholder values. This is possible due to: (i) the premise that engineering work is located mostly at the level of design requirements; and (ii) the expectation that investigating how design requirement relate to norms and values will allow taking stock of the nuances of biosafety as a changing value. Values can have multiple interpretations, and norms can be formal and informal and capture the dominant way of doing things in a community. While we do not follow a VSD approach to identify stakeholder values, we structure our findings using the VSD framework as described by Friedman and colleagues (2013) by presenting our findings in terms of conceptual, empirical, and finally technical investigation into the value of biosafety. Another advantage of choosing the VSD framework to present our work is that it broadens the scope of stakeholders to consider by including direct stakeholders, *i.e*., those who have a say and interact with the technology, and indirect stakeholders, *i.e*., those who will be impacted by the technologies. This widens the scope of the work of the designer.

We ask: who are the relevant stakeholders in the choice of design requirements for biosafety (conceptual)? How do their understandings of norms influence the understanding of the value of biosafety (empirical)? What kind of limitations do these norms impose on design choices (technical)? Taken together, how does this analysis inform the concept of value change?

While recent scholarship presents methods for multi-stakeholder dialogue specifically for biosafety, these are not directly anchored in the concepts of VSD and value hierarchy that we take for our investigation. There are similarities to be found however. For instance, VSD has under its toolkit card-based instruments like “Envisioning Cards” (Friedman & Hendry, 2012; Winkler & Spiekermann, 2021), and beyond that, the recently developed “Cards for Biosafety” from the TU Delft GameLab (Freese et al., 2022) could fit within the broader VSD framework and potentially be a tool in designing for the changing value of biosafety. In addition, new approaches for tracing value change have been published in the context of other emerging technologies, including quantitative use of text corpora (de Wildt et al., 2022) and ex ante assessment of social acceptance (de Wildt et al., 2021), which brings forward the diversity of approaches to take stock of changing values. While we use VSD to make sense of our data, and not to design our technology, we find specially interesting the approach of Umbrello and van de Poel (2021), in which the unique challenges of a given technology (in their case, AI systems) motivate the revision of the VSD process in order to accommodate these specific questions. We come back to their approach in our final section.

Whereas other research projects have aimed to explore different perspectives or approaches to assess safety in biotechnology (Bouchaut & Asveld, 2020, 2021; Bouchaut et al., 2022), or design options for biocontainment (Arnolds et al., 2021; Whitford et al., 2018), this paper is comparatively unique in that it investigates biosafety as a changing value and at the same time provides insights on mechanisms related to value hierarchy for value specification that play at the level of norms and impact the choice of design requirements.

Herein, we show that tensions between stakeholders occur at the level of norms. Formal and informal norms present a crucial component of the value hierarchy that needs to be addressed when designing for value change. We discuss this by first reviewing important aspects of VSD research, then we present results of qualitative interviews with practitioners in biosafety and IB, and finally we discuss stakeholder norms for biosafety, reasonings about genetic safeguards, how these impact the practice of designing for biosafety, and what this entails for designing for value specification.

## Methods and Findings

Designing for biosafety is akin to safety engineering (Doorn & Hansson, 2015) as it also delivers design options for safety like different types of barriers, of which genetic safeguards are the most recent and illustrative exemplar. In order to investigate the changing value of biosafety in IB and the existing design options, we performed desk research and qualitative interviews.

This section successively presents methods and results for conceptual, empirical and finally technical investigations.

### Conceptual Investigations: Identifying Peripheral Issues to Genetic Safeguards

#### Methods

For this section, an exploratory qualitative approach was adopted to uncover the different stakeholders that interact with genetic safeguards and to investigate their particular stakes, roles and positions towards the technology. For the purpose of this study, stakeholder mapping was performed using literature review through a key word analysis of related scientific articles in combination with previous experience derived from workshops within the SafeChassis research project. To further enrich the mapping, stakeholders were categorized into direct or indirect according to those who make decisions and those who are affected by the decisions made.

#### Results

Our research finds a complex network of direct and indirect stakeholders who are positioned around the concept of designing for biosafety. Direct stakeholders, namely researchers, regulators, risk assessors, policy makers and industry, perceive the technology of genetic safeguards in different ways, which do not always align with each other or with the mainstream academic discourse found in the literature (as argued more generally for SbD in Bouchaut & Asveld, 2020). First, biotechnologists and synthetic biologists envision and design genetic safeguards as creative ideas that might or might not be applied but are deemed capable of effectively addressing a given biosafety concern in the laboratory setting (*i.e.*, limit growth of an engineered strain or prevent horizontal gene transfer). An extensive collection of proof-of-concepts has been produced by researchers (Whitford et al., 2018) but, despite technological advances, the integration of these tools into SbD strategies is rarely executed (Robaey, 2018). Second, regulators, risk assessors and policy makers can use technological designs, safeguard research and lab and field studies to collect data and information for risk assessment, establishment of metrics and policy making. Meanwhile, the integration of these designs and tools in regulation remains hypothetical and most probably will ultimately be context-specific and not universal (Kallergi et al., 2021). Lastly, industry tackles physically contained applications with currently sufficient infrastructure and regulation, which makes genetic and biological isolation appear redundant for these settings. Non- or semi-contained applications of genetically engineered agents remain theoretical and, therefore, they are still not contemplated for industrial risk assessment (Asin-Garcia et al., 2020).

While the aforementioned stakeholders have an influence or an interest in the design, development and application of the technology, they coexist with two broad categories of indirect stakeholders: the public and the environment. As part of the public, we encounter civil society. Despite little evidence of its position on genetic safeguards for biosafety, there are concerns expressed about the use of genetic modification in IB (Wouters & Rerimassie, 2017). In some cases, there is stark opposition against an IB innovation (Asveld & Stemerding, 2017; ETC Group, 2014), while in other cases, the social debate around biotechnology and SynBio concentrates precisely on the balance between the technology’s benefits and the biosafety (Bouchaut & Asveld, 2020; Schmidt, 2008). Taking into account biases and factors of cognitive or even sociocultural nature, the public is still the group that bears the potential biosafety risks, specifically for workers, and for human health in general (Merad, 2020; Trump et al., 2018). Yet, the impacts of genetic safeguards and other potential biosafety designs are difficult to assess because the early technical designs generally do not resemble the version that will ultimately be adopted in the final application context (Trump et al., 2020).

In IB, genetically engineered organisms are meant to be physically contained in bioreactors, semi-contained or non-contained at all. The impacts of intentional and the risks of unintentional release position the environment as an indirect stakeholder. Upon release to non-permissive environments, engineered microorganisms are historically not expected to survive in the long run due to their laboratory domestication which likely results in a lower ecological fitness and higher vulnerability to competitors and predators (Cases & Lorenzo, 2005). Nonetheless, these engineered microorganisms might live long enough to transmit engineered DNA to other microbes altering the genetic structure of the ecosystem. This horizontal gene transfer phenomenon represents not only an ecological hazard but could result in the generation of “superbugs” carrying antibiotic resistance genes in nature (Wang & Zhang, 2019).

The risk assessment of potential environmental impacts of engineered microbes will depend on the particular engineered function, the equipped genetic safeguard, and the context of application. The complexity of biotechnology and synthetic biology, together with our limited understanding of natural microbial communities and ecosystems, bring about an enormous degree of uncertainty, which in itself also constitutes a critical challenge (Warner et al., 2020).

This first step of conceptual investigation into the use of genetic safeguards for biosafety in IB underlines that all stakeholders are engaging with the value of biosafety, or are concerned by its absence. In addition, while genetic safeguards are a technology that mostly remains within the confines of academic research, they do seem to provide solutions for potential risks for indirect stakeholders like the public and the environment (Table 1).

**Table 1:** Overview of stakeholders surrounding the biosafety technologies based on the conceptual investigations

Stakeholders		Relation with biosafety technologies	Challenge
Direct	Researchers (biotechnologists, synthetic biologists)	Development, proof-of-concepts to verify hypotheses	Research does not continue beyond laboratory settings
	Regulation and policy organisms (regulators, risk assessors, policy makers)	Source of data for risk assessment and, ultimately, policy making	Integration in real applications is not executed
	Industry	Compliance with regulation	Current regulation is already covered by current infrastructure
Indirect	Public (civil society, workers)	Balance between technology’s benefits and harms (potential health problems)	Difficulties to assess the risks given the underdevelopment of the technology
	Environment	Balance between technology’s benefits and harms (HGT, hazardous “superbugs”, etc.)	

### Empirical Investigations: One Value of Biosafety, Several Meanings

#### Methods

In this empirical investigation, we investigate stakeholders’ views about biosafety in IB, with a focus on practitioners in the biotechnology and SynBio fields. We interviewed representatives of industry (9), academia (4), regulatory bodies (2) and technology transfer experts (2) from May 2019 to June 2021 with two researchers present. Interviewees were recruited considering their experience (senior position within the company/institution) and professional domain. The final list of participants (Ntot = 17) included companies based in the European Union or the USA from a variety of sectors (pharmaceutical, food, chemical or production organism development industries), academics from 4 European universities (from Germany, the Netherlands, Spain, and Switzerland) whose expertise lies in microbial biotechnology and who are engaged in collaborations with multiple industrial sectors, and regulatory bodies and offices from the Netherlands and Denmark (Table 2). After having interviewed approximately half of our participants, the content of the provided answers started to show overlap with previous interviews indicating data saturation. In addition, we found consistency and complementarity of our results with recent research in stakeholder perception of SbD in biotechnology (Bouchaut & Asveld, 2020), which led us to consider our list of participants a representative sample for our exercise. In this phase of the study, we focused on the direct stakeholders with a relation to the industrial context.

**Table 2:** Overview of stakeholder groups with their expertise and location

Sector	Area	Location		Total
		Europe	USA	
Industry	Pharmaceuticals	1	1	2
	Food	1		1
	Chemicals	1		1
	Development of production platforms	1	1	2
	Combination	2	1	3
Academia	IB and SynBio	4		4
Public sector	Regulation	1		1
	Policy making	1		1
	Technology transfer	2		2
				17

The interviews followed a semi-structured approach with open-ended and general questions to prevent bias and to allow for a more in-depth discussion and included questions focusing on the concepts of genetic safeguards’ implementation and utility, SbD, and perceptions of risks and uncertainty. In addition, the use of appreciative inquiry (Cooperrider et al., 2003) in the questions encouraged and inspired the participants to answer according to their own perspectives, ideas and experiences, as opposed to following a strict interview structure. With the prior verbal and written consent of the participants, all interviews were audio-recorded for subsequent transcription (intelligent verbatim style). In order to eliminate bias, transcripts were pseudonymized and encoded with a letter (I = industry, A = academic, R = regulation, policy or technology transfer representative) and a number, before data analysis which was later performed using QCA map (Fenzl & Mün, 2017) and following the six steps for qualitative data analysis (Braun & Clarke, 2006). Based on predefined top-down codes and bottom-up codes derived during the transcription of the interviews, three main themes were formulated: (A) meanings and norms of biosafety; (B) reasoning about genetic safeguards; and, (C) implementation of genetic safeguards.

#### Results

##### Meanings and Norms of Biosafety

The value of biosafety has shown many different meanings throughout the course of our investigation. This plurality soon became apparent from the norms ascribed by our participants to biosafety. In Table 3 we present our findings of four main norms of biosafety: (I) Compliance with regulation; (II) Evaluation of microbes; (III) Responsibility; and, IV) Assessment of risk and uncertainty.

**Table 3:** Main norm groups representing the different stakeholders’ views of the value of biosafety together with the corresponding design approaches. Each group is accompanied by a representative quote and sources supporting or stating each of the design approaches are shown in the last column with a numerical code (I = industry, A = academic, R = regulation, policy or technology transfer representative)

Norms	Design approach	Description	Source
**Compliance with regulation:***“The physical containment is sufficient to comply with regulations, and that is the key thing, we need to comply with regulations.”* (I2)	Biological containment	Use of hosts microorganisms with a reduce host range, with natural or genetically modified characteristics that diminish their invading capacity or virulence, self-inactivating vectors, etc.	I3, I4, I6, R1, A4
	Physical containment	All the physical barriers that prevent or minimize the escape of the microorganisms from the controlled settings	I2, I3, I6, R2, A4, I8
	GMO-free products	Separation of producer and products and inactivation of the biomass	I3, I4, I5, A2, R1, I8
	Historical argument of biosafety	Engineered strains retain the biosafety category granted to their ancestors	I2, I7, A3, I8, I9
	Regulation	Biosafety committees that take care of specific controls and standards. Additional approvals and bigger dossiers than other bioprocesses	I2, I5, I6, I7, A3, R1, R2, I8, I9, R4
**Evaluation of microbes:***“The whole thing is that you should really study your microorganisms carefully and monitor things and be aware of what could happen.”* (A4)	Study of introduced genetic elements	Monitorization of stability and mobility of introduced genetic elements	I3, I9
	Sequencing	Sequence check of plasmids and full genome	I4, I5, I6, I7, I9
	Other assays	Growth, productivity and fitness assays	I5, I7, I8, I9
**Responsibility*:****“That's why the Safe-by-design concept try to promote a proactive approach by actors so that the government doesn't need to solve problems afterwards, but that the actors who develop something, who innovate, who develop a new technology or new application, think about the safety aspects during that process.”* (R2)	Multi-actor responsibility	Proactive responsibility at all stages of the process. Safe-by-design framework	I4, R2, R3
	Cellular barcoding	Accountability through identification of labelled cells through space, time and even cell division which allows the instant access to all the information associated to a particular construct including its origin, its nature, if it is sensitive to antibiotics, what countermeasures one could take, etc.	A3, I9
**Assessment of risk and uncertainty:***“If we work in a reasonably well-established organism like* E. coli *or* Saccharomyces*, I would say that we do not do any test to see whether it's safe or not. We basically go off the literature where others have done those tests already.”* (I8)	Domestication	Human selection of strains to obtain cultivated variants that thrive in artificial niches and meet specific requirements. During this process, microbes become more efficient in consuming particular nutrients, coping with research- or industry-specific stress factors, and producing the target compounds, but this usually comes at the cost of a dramatic decrease of fitness in their natural environment	I4, I6, I9, R1
	Scientific uncertainty	Uncertain risks beyond the imposed norms and extra measures	I2, I4, I6, A3, A4, R2, I8, R3
	Non-fitting assessment	Current regulation does not cover all the aspects of the technology	A2, R2

The norms collected in Table 3 reflect the plurality of the different understandings of biosafety encompassing categories that sometimes do not even vary along the same underlying dimensions (some of them are rules, some are individual practices, and some are general assumptions). While most of them could be considered complementary and particular facets of the value, some of them appear contradicting, which originates the possibility of value tensions (*e.g*., historical argument of biosafety vs. scientific uncertainty).

Genetic safeguards are not found in our empirical investigation. However, one could envision genetic safeguards as part of the biological containment (norm of compliance), or as part of multi-actor responsibility by giving deliberate attention to biosafety since the conception of a strain’s engineering (norms of responsibility).

##### Reasoning About Genetic Safeguards

Stakeholders reason differently about the need of genetic safeguards as design requirements for biosafety. Table 4 collects the main arguments presented under the following aspects identified from our analysis: I) Influence of stakeholders on industrial GMOs; II) Incentives of industry; and, III) Missing elements for safer IB.

**Table 4:** Subthemes affecting the establishment of stakeholders’ norms with their respective supporting arguments. Each subtheme is accompanied by a representative quote and sources supporting or stating each of the arguments are shown in the last column with a numerical code (I = industry, A = academic, R = regulation, policy or technology transfer representative)

Subthemes	Arguments concerning	Arguments	Source
**Stakeholders’ influence on industrial GMOs:** *“The applications of industrial biotech are so broad, and they’re increasing. They’re coming out [from so many] different areas, different sectors, *etc*. that there are different situations. And the rules are changing. You know, as public perception changes, and people become more comfortable sometimes with the guidelines.”* (I5)	Public	Uses industrial GMO products daily	I4, I5
		Perceives risks in GMO products	I2, I4, I5, R1, I9, R3
		Demands GMO-free products	I3, A2, R1, R3
		Responds to marketing campaigns	I6, R2, R3
		Thinks that the technology should be more understandable and simpler, as well as transparent, communicative and honest	I4, A3, R1, R2
		Argues that more biosafety might imply that the former products were not safe	I7
	Environmental	Naturalness argument (natural is more desired than engineered)	I3, I8
		GMO products are more sustainable	I3, I8, R3
	Specificity	Territorial differences: GMOs are better seen and less regulated in USA than in Europe	I5, I6, A2, I8, R3, R4
		Industrial field differences: GMOs are better seen in chemical industry than in health and food industries	A1, A2, R3
	Regulation	Makes a balance between harms and benefits	A1
		Dictates what products reach the market	A3, R2, R3
		Needs to track responsibility	A3, R2
		Encounters secrecy and a non-continuous dialogue with industry	R2
	Other companies	IP prevents the use of some techniques	I4
**Incentives of industry:** *“Their difficulties are ‘what do I need to deliver in order to get the approval of being undertaking that activity in my plant?’ ‘How much time and effort does it cost in the Netherlands, and is it worthwhile setting up a plant here, or should I do it abroad where perhaps it costs me less information and less money and the permits are given earlier?’ because, in R&D, time is of the essence because the competition is everywhere.”* (R2)	Marketing	New markets	I2, R2
		Good image	A2, R2, R3, R4
		Communication, proactivity and honesty	I4, R2
	Production	Cheaper costs	I2, I3, R1, R2
		Minimal alteration of the production chain	I2, I7, A4, I8, R3
		Better (or at least the same) titers, rates and yields	I4, I6, A4, I8
		More robust strains and with better lifestyle	I7, A3
		Sustainable bioprocesses	I4, I6, R2
	Safety	Intrinsic safety	I4, A3, R2
		Broader view in risk assessment	A2, R1
		Tracing responsibility (barcoding) to prevent industrial espionage	A3, I9
**Missing elements for safer IB:** *“I think that safety for release of environmental bacteria have to first overcome the problem number one: how to put them in an environment that works. And when it works, we can discuss about safety issues. But let's face it, the limitation has never been the prohibitions or the regulations for their release, the limitation has always been the efficacy.”* (A3)	Technical part	High-throughput and automation	I2, A3
	Communication	Education of costumers	I3, R2, R3
		Rebranding of GMOs	I3, R3
	Regulation	New, more fitting regulation	A1, R2
		Holistic approach	R2
	Safety	Genetic containment	I5, I7, R1, R2
		Further research on genetic containment and gap analysis	I5, I7, R1, R2, A4, R3
		Biosafety metrics	A3, R2
		Successful stories	A3, R3, R4
		Barcodes and elements of responsibility	I6, A3, R2, I9
		Prudence	R2

##### Implementing Genetic Safeguards

**Fig. 1:**
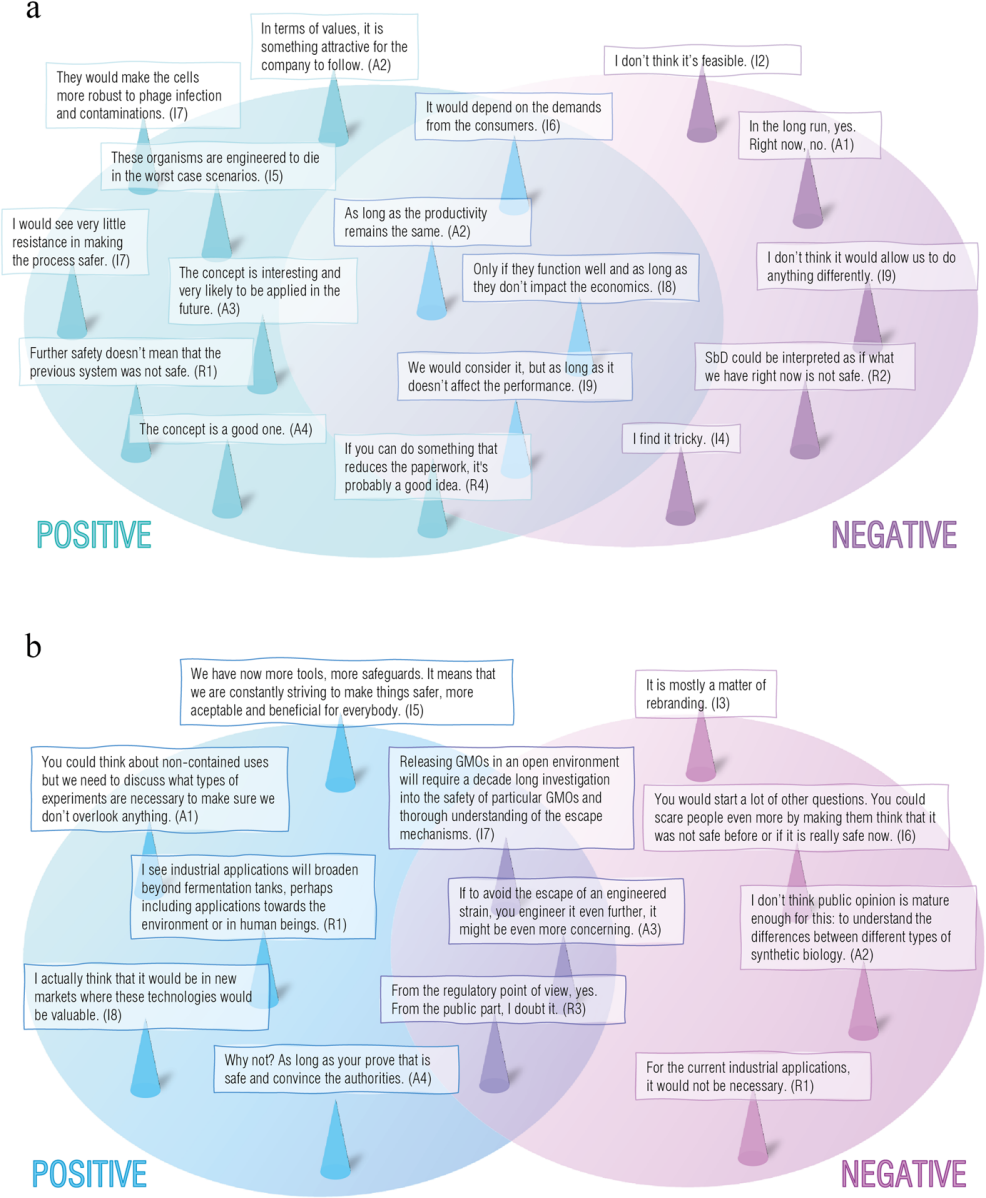
Venn diagrams containing participants’ views on suitability of feasibility of genetic safeguards (**a**) and the opportunities of this technology (**b**) in the field of IB. The schematic overview represents the positive opinions in the left circles and the negative considerations in the right circles, while mixed aspects are found in the overlapping regions. Each statement has been directly obtained or simplified from a representative quote whose source is indicated with a numerical code (I = industry, A = academic, R = regulation, policy or technology transfer representative)

To determine the position of genetic safeguards in the stakeholders’ ecosystem, participants were asked about the suitability and feasibility of implementing genetic safeguards in IB (Fig. 1A), and about the new opportunities that they could bring (Fig. 1B). When it comes to implementation, we find overall mixed positions on those issues, ranging from positive to neutral and negative.

Overall, our empirical findings highlight heterogeneous ascriptions to different norms of biosafety, to reasoning about biosafety and to implementing genetic safeguards. This is striking because when it comes to biotechnology, certain stakeholder groups are typically associated with certain views on safety. This is, for example, the case of the stereotypically concerned and scared public, the very strict regulators, or the careless corporations blinded by the money.

### Technical Investigations: Biosafety, a Value Ready to Change

#### Methods

In this section we analyze the properties of the genetic safeguards as technical measures to address the value of biosafety. For this purpose, we reviewed the scientific literature on the existing technology and connected the known technical features with the value. Moreover, we relied on our background knowledge in addition to the gathered information from literature to pinpoint what the limitations to support the committed value are and how these could be resolved though new biosafety design requirements.

#### Results

Since the second half of the XIX century, biosafety practices have been implemented in response to potential risks associated with the exposure to microorganisms cultured in laboratories. Over the years, more and more protective measures against biological risks have been developed and adopted, typically combining physical containment, working practices and personal protective equipment, and focusing mainly on occupational safety (Beeckman & Rüdelsheim, 2020). With the advent of recombinant DNA and cloning, experts started recommending an extra layer of biological containment on top of the previous barriers (Berg et al., 1975). Shortly after, the U. S. National Institutes of Health (NIH) published the first “Guidelines for Research Involving Recombinant DNA Molecules” (U.S. National Institutes of Health, 1976) which worked as a baseline for many of the present regulations on contained use.

In this paper, the focus of the technical investigations lies on the scientific properties of those biological and genetic layers of containment and their underlying mechanisms related to the value of biosafety. As we have mentioned before, the widespread view is that these built-in safety genetic safeguards can satisfy regulatory demands by reducing the risks and uncertainty of biotechnology and SynBio. This concept however remains mostly speculative given the limited experimental data to support or refute the notion (Rycroft et al., 2019).

Risk assessment and reduction of risks should be considered as quantitatively as possible. When talking about safety, the issue of thresholds and levels within certain ranges becomes important. Quantitative data would not only provide objectivity but would also be a source of both a more comprehensive understanding of the system and a more tangible guidance for policy making. This would be achieved not only by using the appropriate containment tools but also with a series of well-supplied quantitative metrics and robust assessment methodologies. However, these remain scarce. At present, risks are typically considered qualitatively, where the probability of an adverse outcome is expressed as more or less likely than a comparative scenario (*e.g.*, wild type comparators) (Committee on Future Biotechnology Products and Opportunities to Enhance Capabilities of the Biotechnology Regulatory System et al. 2017). Special mention should be made of the evaluation of the escape frequency for a given genetic safeguard. Escape frequency is, together with the strain fitness, one of the few quantitative metrics available and the most popular across the biocontainment literature. It expresses the probability that a microbe equipped with a genetic safeguard has of escaping the permissive conditions. Nonetheless, the detection limit to assess escape frequencies is currently not low enough and it would need to be significantly lower for assessing genetically engineered organisms that are not intended to be physically contained (*e.g.*, intentional environmental release or applications within the human body).

Added to this, metrics should be international and standardized in order to provide sufficient incentives to commit the resources required to achieve high levels of biosafety in laboratories, companies and institutions. (Kwik Gronvall, 2017; Kwik Gronvall & Rozo, 2015; Scientific Committee on Health and Environmental Risks (SCHER) et al. 2015). Data cannot be compared if they are obtained in different media or different contextual circumstances, which means that beyond further metrics, standard protocols to implement them and conduct them are also necessary (de Lorenzo et al., 2021). Furthermore, it is practically impossible to test and validate beforehand safeguard strategies for every conceivable condition or application setting, which implies that assessment of their safety must be, in a higher or lower degree, contextual (Kallergi et al., 2021). This early focus on context should reduce the infinite test conditions to an attainable number, which would likely be still large, given that any ecosystem is, by nature, dynamic. The key to address this point is developing technology to test genetic safeguards in a very large number of environmental conditions. Lorenzo and Schmidt proposed as a solution the generation of thousands of microenvironments in small droplets. By making use of micro and milli-fluidics devices, one could create a myriad of scenarios with specific and fluctuating parameters (oxygen concentration, temperature, pH, humidity, etc.) to test engineered organisms equipped with genetic safeguards in a high-throughput manner. Even though complete ecosystems cannot be completely mimicked in a small drop, this first approach could be the source of copious amounts of valuable information (de Lorenzo et al., 2021).

These developments indicate that biosafety has the potential to be redefined at a technical level, indicating a case of value specification. This section also underscores the limitations of using these technical measures, which also require more research attention.

## Discussion

### The Importance of Norms in Value Change

In this research, we identified a tension between the renewed interest in available design options procured by the SynBio field for biosafety presented in the introduction and their apparent potential for implementation (Fig. 1). By considering and applying the notion of value hierarchy (van de Poel, 2013) to biosafety, we honed in on the sources of this tension, which we found located at the level of norms. While the conceptual investigation underlined the interest for genetic safeguards, the empirical investigations uncovered a heterogeneous understanding of various norms of biosafety independent of membership of specific stakeholder groups where the role of genetic safeguard was yet to be defined. We clustered these norms in four categories, namely compliance with regulation, evaluation of microbes, norms that attend to responsibility, and assessment of risk and uncertainty (Table 3). We can further analyze these norms in terms of formal and informal norms. For instance, “Compliance with regulation” encompasses formal norms codified in laws and regulations. The other three groups of identified norms mostly consist of informal norms that are sometimes used, but not consistently across stakeholders, and without being formally codified. This results in disparities. For example, the monitoring of the uncertain aspects of engineered organisms collides with the idea of domestication. While the former aims at considering unknown risks, the latter reasons that industrial strains are not fit for proliferation out of the laboratory setting and therefore not immediately concerning. In the same line than domestication, we find the historical argument of safety that allows engineered strains to retain the biosafety category of their ancestors despite the many unknowns associated to this practice. Later in the technical investigation and the results depicted in Fig. 1, it became apparent that for value specification to happen in practice, there must be a prior stakeholder alignment.

The tension we identify can therefore be explained by formal norms that may stifle the possibilities for novel design requirements that stem from informal norms of biosafety practice. This tension at the level of norms brings technical innovations at odds with societal needs. Biotechnological innovations are developed to address major societal needs (global warming mitigation, human and animal health solutions, etc.), and biosafety is generally understood as the boundary that prevents these biotech innovations to go through. Thus, society is missing certain socioeconomic benefits derived from biotechnological applications with engineered microorganisms beyond containment because of the lack of roadmaps to assess their biosafety levels or biosafety strategies. Formal norms contribute mainly to current regulatory compliance, instead of making space for other stakeholder norms found in practice. This affects the diversity of views that can get as fundamental as the own orientation of the value. For most, the strains would be generally perceived as the risk actor of the discussion, whereas some stakeholders switch the direction and interpret them as the sensitive element subjected to the risks (industrial espionage, trade secret, and property), what would certainly derive in very different design requirements. Moreover, elements of responsibility will differ as well depending to whom this one is attributed, whether it falls to the developer or to all actors involved.

When designing for a value like biosafety, our findings indicate that inclusion and alignment of stakeholders should be considered as an essential part contributing to the flexibility that van de Poel presented as one of the technical features that allow to better deal with value change, next to adaptability and robustness (2018). Flexibility is understood in terms of different possibilities for using a design. In an absolutely predictable situation, a designer would not need or want the design to be flexible as its function and the values that it is to meet are known. However, when dealing with non-predictable scenarios and changing values like biosafety, flexibility is imperative as it is the involvement of relevant stakeholders to help envisioning how to better meet certain values in the new circumstances (van de Poel, 2018). Complementary to the necessary but inflexible formal norms, other stakeholder norms become indispensable to refine the use of the technology for a changing value.

Designing for biosafety is an exemplar for value specification as understanding of risks expands and adjusts according to the increasing demands for biotechnological innovations. As a consequence, the concept of value specification could be refined in situations like this one as value re-specification as it seems to be leading to potentially new specifications rather to an existing one. Current trends in the field such as cultures of microbial communities (Peng et al., 2021), accelerated evolution and genetic diversification (Simon et al., 2019), and a shift towards robust non-traditional chassis (Martin-Pascual et al., 2021) step away from the classical definition of domesticated microbes and call for a more holistic approach regarding safety. Additionally, most of the norms depicted in Table 3 refer to an IB that is carried out in physically contained settings. However, the prospects of SynBio and IB incorporate applications beyond the bioreactor, including non-controlled environments such as the open field or the human body. One can expect that as scientific knowledge on GMOs increases, awareness and judgement of how to reach safe scenarios will also change accordingly.

### Norms and Reasoning About Genetic Safeguards for Biosafety

This study emerges as an initiative of SynBio designers to reflect on the goal of the biosafety research carried out within the Dutch TTW funded project SafeChassis. Designers often find themselves not only having to design for competing values (van de Kaa et al., 2020), but also dealing with competing stakeholder norms as highlighted in the previous section. In this section, we identify at least three main ways in which reasoning about biosafety impacts norms and complicates taking action for designers: influence of public perception, competing values, and practicability. Finally we reflect on these mechanisms of value change.

A first instance of reasoning lies in the role of public opinion. Public perception is the main pillar of the marketing campaigns and hence prevention of a negative image ends up being one of the most important drivers during the design of a business model in IB (Table 4, Fig. 1). Previous studies on opinion towards GMOs showcase that certain events or scandals might impact public opinion in the long term and affect later policy decisions (Scientific Committee, 2020; Ferretti, 2007; Jr & Durant, 2010). Arguments concerning the public on Table 4 also illustrate the general belief that the public perceives risks in GMO products and consequently demands GMO-free products. However, some more recent studies report that public views do not always align with experts’ opinions and that editing technologies are sometimes portrayed as positive (Marcon et al., 2019; McCaughey et al., 2018). This, of course, depends on different factors including the cultural context and the type of industry and product. While the stigma around GMOs is greater in Europe than it is in the United States, the use of engineered microorganisms for bioproduction in the field of IB is equal in the two regions, despite the a priori different legislations and norms. Nonetheless, more considerations besides the territory arise as one gets closer to anything that approaches consumer products. The use of GMOs for the production of chemicals is perceived as better than their use for medicines and drugs, and this one would in turn be better ranked than their use for food production. This implies different obligations and norms for biosafety for different use cases of GMOs (Table 4, Stakeholders’ influence on industrial GMOs, Specificity).

A second instance of where norms of biosafety are impacted concerns reasoning in relation to value prioritizations, where depending on arguments and views, new genetic safeguards can either be dismissed, or encouraged. Arguments such as naturalness, risk and uncertainty have historically been used to justify rejection towards GMOs (Sandin & Robaey, 2020), whereas sustainability, innovation and an overall positive balance of benefits versus harms have played the opposite role. In the matter of genetic safeguards, the negative and neutral quotes presented in Fig. 1 showcase how the application of this technology can be simply deemed unfeasible or even superfluous when the process is already considered safe enough. In these grounds, prioritizations of other values such as productivity or economic interests over technologies for biosafety will always be legitimized. Furthermore, and even if these other major interests would remain unaffected, investing in genetic safeguards could be interpreted from two opposite angles. One could either see them as a way to make the process safer, or as a way to make it merely safe, implying that previous processes were not safe in the first place. This argument constitutes the main opposition towards the idea of genetic safeguards. If biosafety would be positioned not as a standalone, but as a complementary value that would benefit other interests such as economic growth and broader applications (Fig. 1B), judgement of the genetic safeguard technology would be adjusted. Genetic safeguards could then cease to appear superfluous and represent the only necessary design requirement for biosafety in non-contained application contexts (Asin-Garcia et al., 2020). In addition, some of these strategies’ features hold the promise of being profitable for other aspects of the industrial process by, for example, decreasing some of the costs (Asin-Garcia et al., 2022; Ma & Isaacs, 2016; Selão et al., 2019), or increasing the production yields (Aslan et al., 2017).

Third and last dynamic, as we present in this research, is the link between technology readiness and reasoning that make that genetic safeguards are not yet widely used in real-world applications despite being a powerful innovation for biosafety. We call this practicability. When assessing reasonings concerning legitimizing their use, participants often mentioned that genetic safeguards first need to be ready to deliver. The arguments concerning missing elements presented in Table 4 and the technical issues described in Table 5 underline the need for further efforts and research for genetic safeguards to be able to yield the expected outcomes towards the value of biosafety. In turn, reasoning that they are needed would help their development from the purely research curiosity to practicability.

**Table 5:** Technical issues for biosafety risk assessment with genetic safeguards

Issues	Current limitations	New biosafety design requirements
Risks are mostly considered qualitatively	Not enough metrics and data for risk assessment	Quantitative or semiquantitative new metrics beyond escape frequency
Data is obtained in different contextual circumstances	No consensus on how to measure biosafety and which are the expected levels	Standardized protocols for assessment
Standard validation should be performed in infinite scenarios	It practical terms, it is impossible to test in infinite application settings	Contextualization (reduction of the number of test conditions) and high-throughput assessment

When considering value change, taking stock not only of different stakeholder norms when it comes to value specification is important. Understanding how stakeholders influence each other in their reasoning about norms is even more crucial. The influence of public perception is often presented as a reasoning for doing biosafety as compliance. Competing values are used to both justify using genetic safeguards, but also not. Lastly, practicability seems to be a bottleneck in stakeholders reasoning. Making these dynamics explicit would be the first step in having a discussion about value specification and the related norms and derived design requirements.

### Limitations

A possible limitation of our research was to look at genetic safeguards broadly in IB, since our results suggest that biosafety design requirements depend on the application setting. Further research should consider designing for biosafety in a context-specific application (Kallergi et al., 2021) that would lead to design changes (Winkler & Spiekermann, 2018) and would consider not only the development of technologies but rather their entire lifecycle (de Reuver et al., 2020). Despite being largely an educational project, this point is exemplified by the iGEM competition teams throughout thorough studies of genetic safeguards in their very specific innovations (Robaey, 2018). This would open the door for further research that would aim at using VSD to improve existing design practices in biotechnology, such as the Design-Build-Test-Learn framework commonly used in the field. In this way further research could provide a similar model than the one proposed by Umbrello and van de Poel (2021) in the context of AI. 

## Conclusions

At the beginning of our investigation we asked: who are relevant stakeholders in the choice of design requirements for biosafety (conceptual)? How do their understandings of norms influence the understanding of the value of biosafety (empirical)? What kind of limitations do these norms impose on design choices (technical)? Taken together, how does this analysis inform the concept of value change?

We found that researchers, regulation and policy organisms and industry are the direct stakeholders dictating the design requirements for biosafety. However, it is the public and the environment who, as indirect stakeholders are ultimately affected by these choices. We discovered that the apparent stalemate in genetic safeguards implementation in industrial biotechnology is motivated by different stakeholder understandings of the value of biosafety which, in turn, derive in a disarray of envisioned norms for the foreseeable applications. This lack of consensus originates technical limitations related to deficiency of standardized protocols and practices, absence of quantitative metrics and data for risk assessment, and out-of-context evaluations. Biosafety is not the same for everybody, and neither it is our judgment of it in emerging situations. The value of biosafety is therefore subjected to value change since its relevance or priority depends on how is exactly understood and how limited is by the technology that supports it.

Due to the identified tensions at the level of stakeholder norms and arguments *e.g*., compliance vs. innovation, sustainability vs. naturalness, uncertainty vs. domestication, responsibility from or towards engineered strains, etc., we suggest that efforts recommended in Responsible Research and Innovation (RRI) to achieve stakeholder alignment (Kuzma & Roberts, 2018) can also benefit when designing for changing values. Since the value of biosafety has significant societal impacts, we consider crucial stakeholder participation during the process to discuss concerns and to formulate alternative solutions. Our research suggests that these insights should also be extended to discussions about biosafety norms in order to address tensions stemming from formal and informal norms, and design for the value, in our case, of biosafety.

## Data Availability

Records and transcripts of interviews will remain anonymous, confidential and saved in a secure network to maximally 5 years after the initial research.
